# Fibrinogen‐binding M‐related proteins facilitate the recruitment of plasminogen by *Streptococcus pyogenes*

**DOI:** 10.1002/pro.70078

**Published:** 2025-03-18

**Authors:** Emma‐Jayne Proctor, Hannah R. Frost, Bhanu Mantri, Sandeep Satapathy, Gwenaëlle Botquin, Jody Gorman, David M. P. De Oliveira, Jason McArthur, Mark R. Davies, Gökhan Tolun, Anne Botteaux, Pierre Smeesters, Martina Sanderson‐Smith

**Affiliations:** ^1^ Molecular Horizons Research Institute and School of Science University of Wollongong Wollongong New South Wales Australia; ^2^ Molecular Bacteriology Laboratory, European Plotkins Institute for Vaccinology (EPIV) Université Libre de Bruxelles Brussels Belgium; ^3^ The Broad Institute of MIT and Harvard Cambridge Massachusetts USA; ^4^ The Institute for Molecular Biosciences, Centre for Superbug Solutions The University of Queensland Brisbane Queensland Australia; ^5^ Department of Microbiology and Immunology, at the Peter Doherty Institute for Infection and Immunity The University of Melbourne Melbourne Victoria Australia; ^6^ The ARC Training Centre for Cryo‐electron Microscopy of Membrane Proteins, University of Wollongong Wollongong NSW Australia

**Keywords:** fibrinogen, Group A *Streptococcus*, M‐related protein, plasminogen, *Streptococcus pyogenes*

## Abstract

Group A *Streptococcus* (GAS) M‐related proteins (Mrp) are dimeric α‐helical coiled‐coil cell‐wall‐attached proteins. During infection, Mrp recruit human fibrinogen (Fg) to the bacterial surface, enhancing phagocytosis resistance and promoting growth in human blood. However, Mrp exhibit a high degree of sequence diversity, clustering into four evolutionarily distinct groups. It is currently unknown whether this diversity affects the host–pathogen interactions mediated by Mrp. In this study, nine Mrp sequences from the four major evolutionary groups were selected to examine the effect of sequence diversity on protein–protein interactions with Fg. Negative staining transmission electron microscopy confirmed that Mrp are fibrillar proteins measuring between 45.4 and 47.3 nm in length, and mass photometry confirmed the ability of Mrp to form dimers. Surface plasmon resonance was used to evaluate the affinity of each Mrp for Fg. All Mrp studied bound to Fg via Fragment D (FgD) with nanomolar affinity. Previous studies have linked the acquisition of plasminogen (Plg) by GAS Fg‐binding M proteins to tissue destruction and excessive stimulation of the human inflammatory response during infection. Our findings show that Mrp provide an alternative mechanism for Plg recruitment, as Plg binding by Mrp was significantly enhanced following pre‐incubation with Fg. These data suggest that Mrp play an important role in GAS host–pathogen interactions. However, further studies are necessary to investigate the relevance of these findings in vivo.

## 1 INTRODUCTION

*Streptococcus pyogenes* (Group A *Streptococcus*; GAS) is a Gram‐positive human pathogen responsible for superficial, self‐limiting infections such as pharyngitis and impetigo, as well as severe invasive diseases including bacteraemia, toxic shock syndrome, and necrotising fasciitis (Walker et al. 2014). GAS infection can also cause autoimmune complications such as rheumatic heart disease and acute post‐streptococcal glomerulonephritis (Cunningham 2000; Walker et al. 2014). Collectively, these conditions are estimated to cause over 500,000 deaths per year, with GAS remaining among the top infectious pathogens contributing to global mortality (Craik et al. 2022). GAS expresses multiple cell surface proteins known to mediate interactions with the host, including the M‐family proteins that include the M protein, the M‐like protein (Enn), and the M‐related protein (Mrp) (Frost et al. 2018). A significant amount of research has focused on the M protein as a key virulence factor due to its high abundance on the cell surface and the widespread presence of the *emm* gene in nearly all GAS strains (Fischetti 1989; Hondorp and Mciver 2007). The heterogeneity of the M protein's N‐terminal region, which is encoded by the 5′ region of the *emm* gene, serves as the foundation for classifying GAS strains using the *emm*‐typing scheme. This method has led to the identification of over 200 distinct *emm*‐types (McMillan et al. 2013).

The gene encoding Mrp is present in approximately 88.5% of GAS strains and is associated with 64.4% of *emm* types responsible for global infection (Frost et al. 2020). Compared to the M protein, Mrp is less variable in sequence, with reports of an average sequence identity of 83.2% between Mrp proteins, compared to 44.7% between M proteins (Frost et al. 2020). Recent analysis of a global collection of GAS isolates identified 221 individual Mrp sequences that could be grouped into four primary genetically distinct clusters and multiple subgroupings based on amino acid sequence variation (Davies et al. 2019; Frost et al. 2023). Mrp were predicted by O'Toole et al. (1992) to adopt a dimeric coiled‐coil fibrillar structure extending 50–60 nm from the GAS cell surface due to amino acid sequence similarities with the well‐characterized M protein (Fischetti 1989; Phillips Jr. et al. 1981). The dimeric α‐helical coiled‐coil nature of Mrp was confirmed using circular dichroism by Cedervall et al. (1997).

Mrp facilitates the resistance of GAS to both the innate and adaptive immune systems through the recruitment of host plasma proteins such as Fg and immunoglobulin G (IgG) (Frost et al. 2018). Recently, Mrp representing all four genetically distinct clusters described by Frost et al. (2023) were shown to bind to IgG with nanomolar affinity, despite having an amino acid pairwise identity ranging from 75.5% to 99.1% (Proctor et al. 2024). This could be explained by minimal sequence diversity within the IgG binding motif of Mrp in the A‐repeat domain. In the case of Fg, the proposed Fg binding domains (FBD) within Mrp are located in the hypervariable N‐terminal domain (Li and Courtney 2011). It is not known whether Mrp diversity within this region affects the interaction with Fg.

Circulating in human blood plasma at concentrations ranging from 1.5 to 4.0 g/L, Fg is a fibrous glycoprotein critical for several biological functions, including hemostasis, wound healing, inflammation, and host immune defense (Weisel & Litvinov 2017). The interaction between Fg and Mrp plays a key role in preventing the deposition of C3b on the surface of M4 GAS, thereby inhibiting phagocytosis in human blood (Li and Courtney 2011). Previous studies have mapped two Fg‐binding domains to the N‐terminus of Mrp from M4 GAS: FBD1, located within amino acid residues 1–55, and FBD2, found within residues 81–138. Both domains mediate the binding of Fg to GAS (Li and Courtney 2011). While FBD2 was confirmed in all 11 Mrp sequences examined by Li and Courtney (2011), FBD1 is not present in all Mrp.

These findings suggest that all Mrp are capable of binding Fg; however, the functional implications of Mrp with secondary Fg‐binding sites remain unclear. Furthermore, Fg binding has only been confirmed in a single Mrp from an M4 strain. Therefore, it is necessary to verify whether diverse Mrp can bind Fg and whether variation in Fg binding exists within the Mrp family. Additionally, the capacity of Mrp to interact with Fg in a complex host environment, such as plasma, where competing binding partners like IgG may be present, is also unknown.

In addition to providing protection against phagocytosis, some Fg‐binding GAS proteins also mediate the recruitment and activation of the host protein plasminogen (Plg) by GAS (Sanderson‐Smith et al. 2012). Plg is a glycoprotein present in plasma and extracellular fluids at approximately 2 μM (Danø et al. 1985). The activation of Plg to the serine protease plasmin (Pln) is central to the fibrinolytic system and facilitates the regulated degradation of fibrin clots, connective tissue, the extracellular matrix, and adhesion proteins in host tissue (Sanderson‐Smith et al. 2012). However, Plg‐binding surface proteins on GAS can hijack the host fibrinolytic system, accelerating GAS dissemination through host tissue via the activation of Pln by GAS‐secreted streptokinase (SK) (Sun et al. 2004; Walker et al. 2005).

Interactions between Fg‐binding M proteins and Plg have been shown to occur through the indirect formation of a tri‐molecular Fg‐Plg‐SK complex. This complex mediates the subsequent generation of proteolytic Pln (McArthur et al. 2008; Sanderson‐Smith et al. 2012; Wang et al. 1995). In blood, digestion of Fg by plasmin produces three distinct domains: FgE flanked by two FgD (Everse et al. 1995; Fuss et al. 2001). The interaction between M proteins and FgD has been shown to enable the subsequent binding of Plg to FgE, thereby facilitating Pln activation. Given that Mrp are well‐characterized Fg‐binding proteins, it is possible that they also support Plg acquisition by GAS; however, this has not yet been investigated.

Considering the prevalence of Mrp in the GAS population, we sought to characterize the interactions between various Mrp and two key host proteins, Fg and Plg, which are critical to GAS virulence. Nine Mrp sequences, representing the four Mrp clusters from a global GAS database (Frost et al. 2023; Proctor et al. 2024), were selected for molecular characterization to assess the effect of Mrp genetic diversity on binding to these host plasma proteins.

We aimed to determine whether the presence of two Fg‐binding domains increases the affinity of Mrp for Fg. Surface plasmon resonance (SPR) experiments showed that all Mrp tested interact with the FgD domain of Fg. Additionally, we found that the presence of a secondary Fg‐binding site within Mrp does not enhance Fg acquisition in plasma. The extent of Fg acquisition from plasma varied among the nine Mrp, regardless of the number of Fg‐binding domains or their affinity, suggesting that more complex binding dynamics occur in the host environment. Host Plg and Pln acquisition was also detected in plasma pulldown experiments; however, only weak interactions between Mrp and Plg were observed in SPR experiments. Nevertheless, pre‐incubation with Fg resulted in increased Plg binding by Mrp; Plg binding assays and SPR experiments confirmed the formation of a tri‐molecular complex of Mrp‐Fg‐Plg.

In summary, these data demonstrate that the Mrp‐Fg interaction is conserved among genetically diverse Mrp and that Mrp may offer an alternative mechanism for Plg recruitment by GAS. This suggests that the Mrp‐Fg interaction may be an important aspect of host–pathogen interactions in GAS virulence, highlighting the need to further characterize these interactions *in vivo.*

## 2 RESULTS

### 2.1 Visualization of Mrp using negative staining TEM

M proteins exist in a dimeric, fibrillar, α‐helical coiled‐coil conformation that extends 50–60 nm from the GAS cell surface (Cedervall et al. 1995; Cedervall et al. 1997; Nilson et al. 1995; Phillips Jr. et al. 1981). Although sequence homology suggests that Mrp might have a similar structure to the M protein, this has not yet been confirmed experimentally. Recently, Frost et al. (2023) identified four evolutionarily distinct clusters of Mrp by analyzing 221 unique Mrp sequences from 1668 GAS genomes. Proctor et al. (2024) examined the binding of Mrp proteins, representing various clusters identified by Frost et al. (2023), to human IgG. Building on this work, this study aimed to investigate the binding of Fg to the same representative Mrp proteins.

**FIGURE 1:**
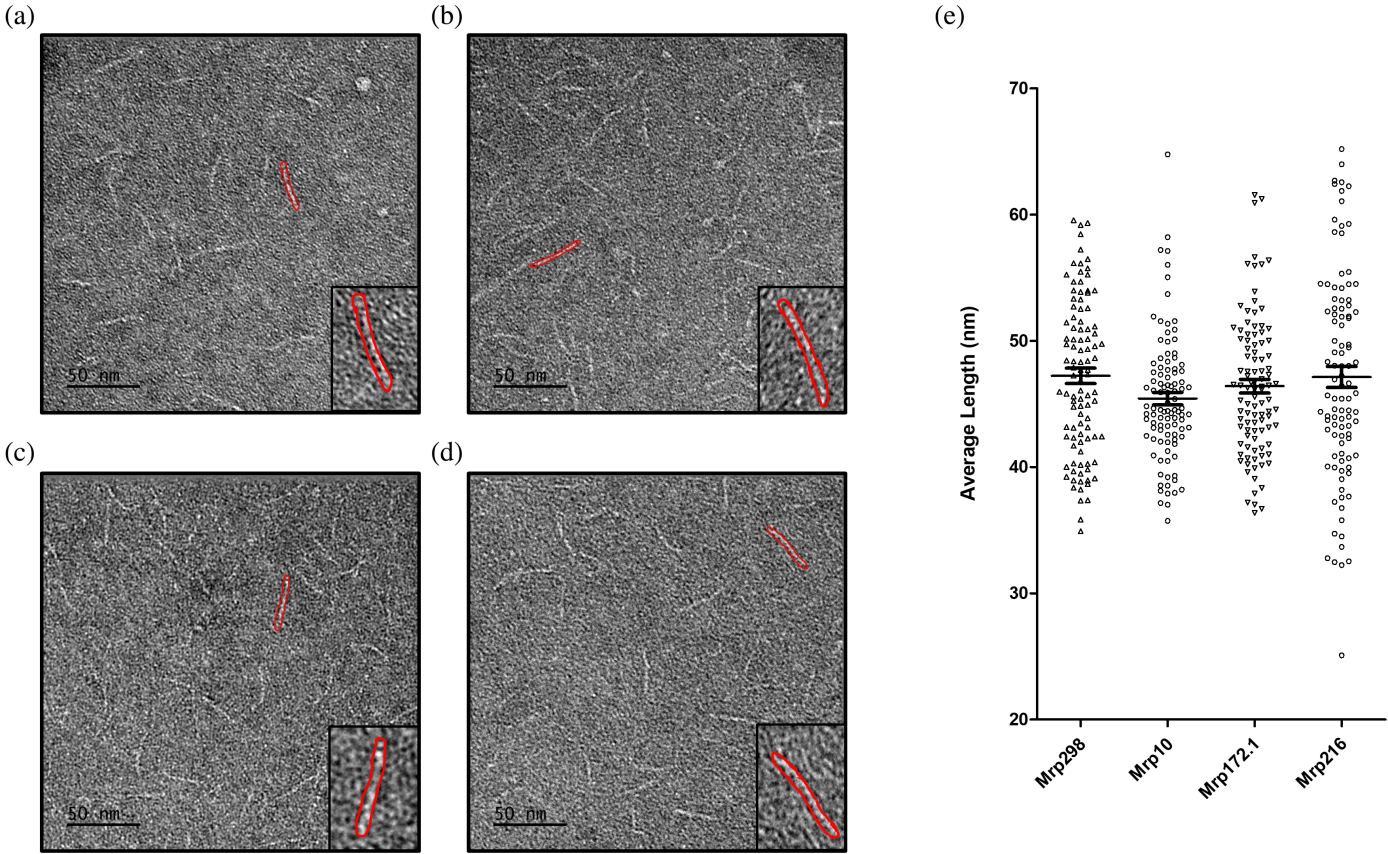
Negative staining TEM confirms fibrillar structure of Mrp representing the four genetic clusters. Representative images show negatively stained samples for (a) Mrp298 from cluster 1, (b) Mrp10 from cluster 2, (c) Mrp71 from cluster 3, and (d) Mrp216 from cluster 4. Scale bar is 50 nm. (e) The average fibril lengths ± SEM for the listed Mrp samples were analyzed (*n* = 100 for each sample) and represented as a graph. No significant difference was seen in average fibril length between Mrps as determined by one‐way ANOVA (*p* > 0.05).

Given the importance of the dimeric structure to Mrp function, we confirmed the ability of recombinant Mrp to dimerize using mass photometry (Figure S1, Supporting Information). We then used negative‐staining transmission electron microscopy (TEM) to visualize proteins representative of each Mrp cluster and calculated the average fibril length for each Mrp (Figure 1). Our data confirmed that Mrp298 (cluster 1), Mrp8 (cluster 2), Mrp71 (cluster 3), and Mrp216 (cluster 4) all adopt a fibrillar structure, with average lengths of 47.3 ± 0.6 nm, 45.4 ± 0.5 nm, 46.4 ± 0.5 nm, and 47.1 ± 0.8 nm, respectively. There was no significant difference in the average Mrp length between clusters (*p* > 0.05) as determined by one‐way ANOVA.

### 2.2 Affinity of Mrp for Fg is not impacted by the number of FBDs

Previously, two FBDs were mapped to the N‐terminus of Mrp4 from an M4 serotype GAS strain (Li and Courtney 2011). The FBD1 (residues 1–55) and FBD2 (residues 81–138) sequences were shown to mediate the binding of Fg to GAS and inhibit phagocytosis in human blood (Li and Courtney 2011). Amino acid sequence analysis of 11 Mrp serotypes confirmed the presence of FBD2 in all Mrp screened, while FBD1 was found in only 6 out of 11 Mrp. In the current study, we performed in silico analysis to examine the conservation of FBDs in the 221 unique Mrp amino acid sequences identified by Frost et al. (2023) (Table 1).

**TABLE 1:** Summary of in silico screen of FBD1 and FBD2 in 221 unique Mrp amino acid sequences identified from 1668 GAS genomes by Frost et al. (2023).

Mrp cluster	Total number of Mrp per cluster	Number of Mrp with FBD1	Percentage of Mrp with FBD1	Number of Mrp with FBD2	Percentage of Mrp with FBD2
1	8	0/8	0%	8/8	100%
2	39	0/39	0%	36/39	92.3%
3	44	0/44	0%	44/44	100%
4	130	130/130	100%	124/130	95.4%

Notably, FBD1 was exclusive to Mrp within cluster 4, accounting for 58.8% (130/221) of all Mrp examined. Conversely, the FBD2 motif was highly conserved across all four clusters, present in 95.9% (212/221) of Mrp sequences. Specifically, the FBD2 motif was found in 100% of cluster 1 (8/8) and cluster 3 (44/44) proteins, 94.5% of cluster 4 (124/130) proteins, and 92.3% of cluster 2 proteins (36/39).

**FIGURE 2:**
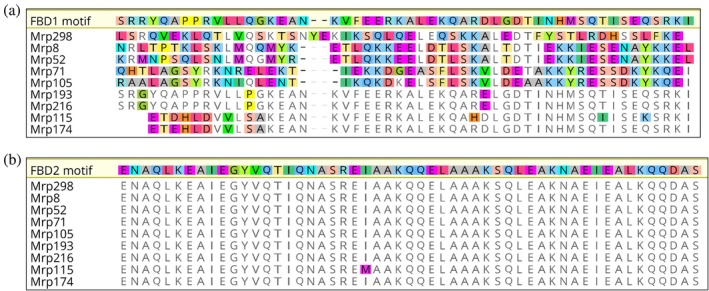
Multiple sequence alignment of Mrp annotated with Fg‐binding domain 1 (FBD1) and Fg‐binding domain 2 (FBD2). Pairwise identity analysis was generated via the pairwise MUSCLE alignment tool using default settings in Geneious (version 6.0, Biomatters). (a) FBD1 and (b) FBD2 were annotated as identified on Mrp4 from Li and Courtney (2011) in the N‐terminal and central core region of Mrp. Mrp298 represents cluster 1, Mrp8 and Mrp52 represent cluster 2, Mrp71 and Mrp105 represent cluster 3, and Mrp193, Mrp216, Mrp115, and Mrp174 represent cluster 4. Amino acids not conserved within the motif are highlighted. Colored amino acids indicate differences between query sequence and reference sequence (FBD1 and FBD2 motifs).

For biophysical analyses, nine Mrp were selected to represent all four clusters and FBD diversity (Proctor et al. 2024). A pairwise MUSCLE alignment was performed, comparing these nine Mrp with Mrp4 containing the canonical FBD1 (Figure 2a) and FBD2 (Figure 2b) sequences. FBD2 was highly conserved across all Mrp in this study (Figure 2b). In contrast, the FBD1 sequence was present in only four out of nine proteins (Figure 2a). Mrp from cluster 4 (Mrp193, Mrp216, Mrp115, and Mrp174) contained the FBD1 sequence of Mrp4 (Figure 2a), while Mrp from cluster 1 (Mrp298), cluster 2 (Mrp8 and Mrp52), and cluster 3 (Mrp71 and Mrp105) did not contain the FBD1 sequence (Figure 2a).

**FIGURE 3:**
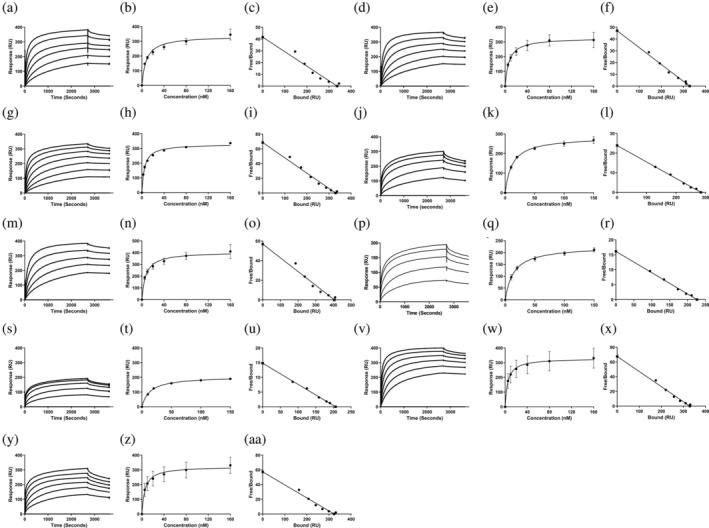
SPR analysis of the interaction between human Fg and Mrp. (a) SPR sensogram generated for the interaction between Mrp and Fg. The steady‐state affinity analysis was produced by the nonlinear fitting of sensorgrams according to a 1:1 Langmuir binding model in BIA evaluation version 4.0.1 (Biacore AB, Uppsala, Sweden). Scatchard plot analysis was conducted in GraphPad Prism 5. Data represent *n* = 3 ± SEM. All assays were performed using Fg in a concentration series of 0–160 nM over a succession of seven separate injections for 3200 s at a flow rate of 10 μL/min followed by a 900 s dissociation period. (a–c) Mrp298, (d–f) Mrp10, (g–i) Mrp52, (j–l) Mrp71, (m–o) Mrp105, (p–r) Mrp216, (s–u) Mrp 210.5, (v–x) Mrp115, and (y–aa) Mrp174.

To determine if the presence of one or two FBDs affects the interaction between Mrp and Fg, we assessed binding using SPR. *K*_*D*_ values were calculated from sensorgrams produced by injecting Fg at concentrations ranging from 0 to 160 nM onto immobilized Mrp (Figure 3 and Table 2). M1, a well‐characterized Fg‐binding protein, and M53, which lacks specific binding to Fg, were used as positive and negative controls, respectively (Figure S2) (Sanderson‐Smith et al. 2014).

**TABLE 2:** Comparison of steady‐state affinities ( *K*_*D*_ in nM) for genetically diverse Mrp with one or two Fibrinogen binding domains (FBD).

Mrp type	Cluster	Number of FBD	Fg (nM)	FgE (nM)	FgD (nM)
298	1	1	7.1 ± 0.3 nM	NB	65.2 ± 2.0 nM
8	2	1	7.7 ± 0.8 nM	NB	94.1 ± 4.1 nM
52	2	1	4.8 ± 0.6 nM	NB	79.2 ± 11.5 nM
71	3	1	7.0 ± 0.2 nM	NB	76.2 ± 1.8 nM
105	3	1	6.9 ± 0.2 nM	NB	88.6 ± 0.6 nM
193	4	2	13.5 ± 0.9 nM	NB	56.8 ± 2.0 nM
216	4	2	14.5 ± 2.2 nM	NB	51.6 ± 1.0 nM
115	4	2	5.8 ± 1.6 nM	NB	84.2 ± 0.8 nM
174	4	2	6.2 ± 1.2 nM	NB	54.4 ± 1.2 nM

*Note*: Data represents the mean ± SEM in nM as determined from three experimental replicates.

Abbreviation: NB, no binding.

The *K*_*D*_ values for all Mrp with Fg ranged from 5.77 to 14.5 nM, indicating that all nine Mrp exhibit high affinity for Fg and that the presence of FBD2 alone is sufficient to mediate this high‐affinity binding (Table 2). However, Mrp216 and Mrp193, which both contain FBD1 motifs, showed a twofold lower affinity for Fg compared to the other Mrp, as indicated by their higher *K*_*D*_ values. Scatchard plot analysis (Weder et al. 1974) of the interactions between all Mrp and Fg produced a linear plot, typical of monovalent interactions. This suggests that a single Fg protein binds to one Mrp, despite the presence of two FBDs in some proteins (Figure 3).

### 2.3 Mrp with reduced Fg binding affinity have FBD1 regions containing longer disordered regions with differences in electrostatic potential

While all Mrp demonstrated affinity for Fg within the nanomolar range, Mrp193 and Mrp216 exhibited a two‐fold lower affinity for Fg compared to the other Mrp in this study. To investigate the possible reasons for this difference in affinity, we compared the amino acid sequences of the FBD1 and FBD2 domains. Notable differences were observed in the composition of the FBD1 domain between Mrp193 and Mrp216 and Mrp115 and Mrp174 (Figure 2). Specifically, the FBD1 domains of Mrp193 and Mrp216 contain prolines and glycine's in the N‐terminal region (Gly4, Pro8, Pro9, Pro14, Gly15 of the mature protein), whereas Mrp115 and Mrp174 do not have these residues (Figure 2a).

**FIGURE 4:**
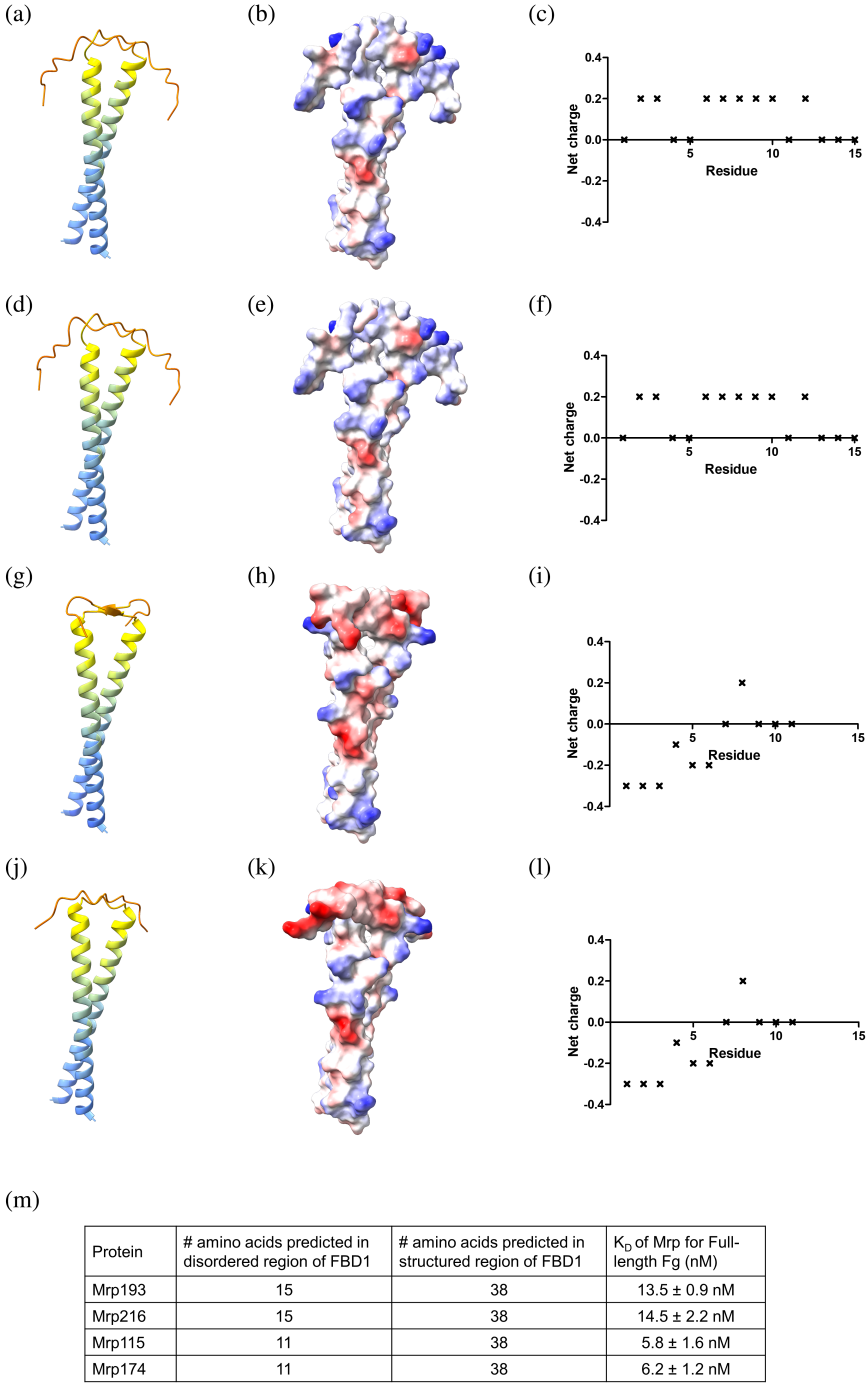
AlphaFold predictions of Mrp variants suggest differences in the lengths and charge of N‐terminal disordered regions. (a, d, g, j) Each residue in the Mrp sequence is color‐coded based on the model confidence score, pLDDT using a lDDT‐Cα metric as per Jumper et al. (2021) and Varadi et al. (2021). AlphaFold predictions suggest that Mrp193 and Mrp216 have longer disordered regions than Mrp115 and Mrp174. (b, e, h, k) Coulombic electrostatic potential was calculated in UCSF ChimeraX 1.3 to visualize the charge at the protein surface. Red represents a negative potential through white to blue for positive potential. (c, f, i, l) The amino acid sequence of each Mrp was analyzed using EMBOSS: Charge (Rice et al. 2000) to predict the net charge of amino acids for the FBD1 sequence of Mrp using a 5 residue (default) window. The predictions were plotted in GraphPad Prism 5. (m) summary of relative portion of structured and disordered amino acids in FBD1 sequence and *K*_*D*_ for interaction with Fg.

To investigate whether differences in FBD1 sequences correlate with structural variations in these Mrp, we generated structural model predictions using AlphaFold (Jumper et al. 2021) for Mrp193, Mrp216, Mrp115, and Mrp174 (Figure 4). A per‐residue confidence score (pLDDT) of <50 is generally considered a strong indicator of disordered regions when the protein is in isolation and is often represented as lines or thin tubes in ribbon/cartoon diagrams. Due to the very low confidence scores, the structure of such regions should not be interpreted definitively (Varadi et al. 2021).

Our predicted structures show regions with pLDDT scores of <50 between amino acids 1–15 for Mrp193 (Figure 4a) and Mrp216 (Figure 4d), and between amino acids 1–11 for Mrp115 (Figure 4g) and Mrp174 (Figure 4j). These regions, which include the N‐terminal portion of the FBD1 domain, may represent disordered regions of Mrp. Notably, Mrp193 and Mrp216, which exhibit reduced affinity for Fg (Figure 4m), are predicted to have longer unstructured regions and consequently shorter α‐helical structures in their FBD1 domains compared to Mrp115 and Mrp174 (Figure 4m).

EMBOSS Charge was used to generate a plot showing the mean charge of amino acids within a 5‐residue window as it moved along the sequence of the FBD1 region. Additionally, the Coulombic electrostatic potential was calculated in UCSF ChimeraX 1.3 for structures generated by AlphaFold to visualize the charge distribution on the protein surface. Mrp193 (Figure 4b,c) and Mrp216 (Figure 4e,f) displayed a net‐positive charge in the unstructured region of the FBD1 domain. In contrast, Mrp115 (Figure 4h,i) and Mrp174 (Figure 4k,l) exhibited a net‐negative charge in the unstructured region of FBD1.

### 2.4 Mrp binds fibrinogen Fragment D

**FIGURE 5:**
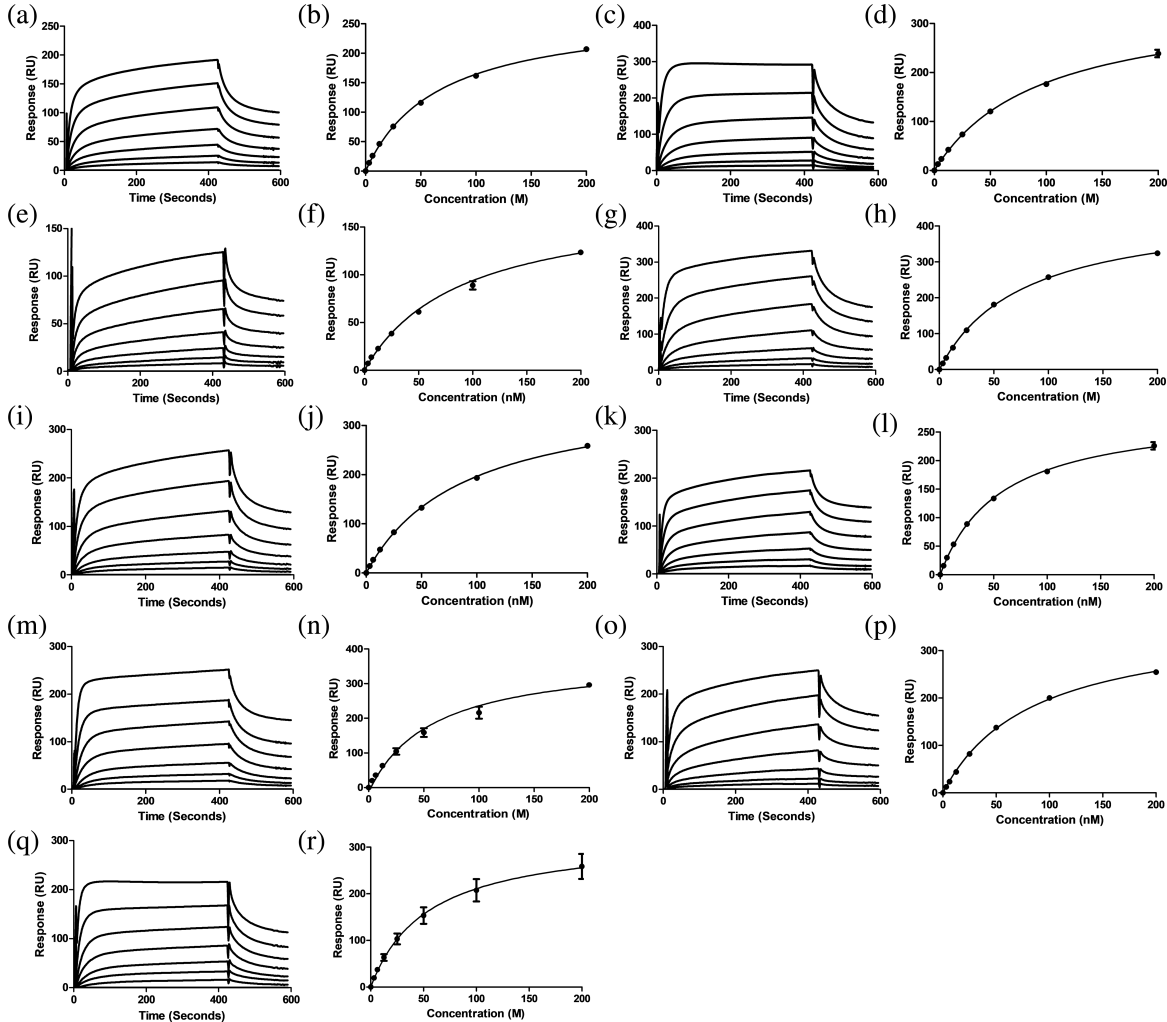
Binding between Mrp and Fg Fragment D (FgD) determined via surface plasmon resonance. Three replicates of sensorgrams depicting FgD at varying concentrations (0–200 nM) over a series of seven separate injections for 420 s at a flow rate of 5 μL/min, followed by a 180 s dissociation period. Steady state affinity plot was used to determine the affinity ( *K*_*D*_ ) of the interaction between Mrp and FgD. Data was analyzed via nonlinear fitting of the sensorgrams according to a 1:1 Langmuir binding model in BIAevaluation (4.0.1) and plotted in GraphPad Prism 5. Data represents *n* = 3 ± SEM. Data represents *n* = 3 ± SEM. (a, b) Mrp298, (c, d) Mrp10, (e, f) Mrp52, (g, h) Mrp71, (i, j) Mrp105, (k, l) Mrp216, (m, n) Mrp 210.5, (o, p) Mrp115, and (q, r) Mrp174.

In blood, Pln digestion of Fg produces three distinct fragments: FgE, flanked by two fibrinogen Fragment D (FgD) domains (Everse et al. 1995; Fuss et al. 2001). These Fg degradation products are abundant at infection sites, and the M1 protein has been shown to bind to FgD, forming pro‐inflammatory networks that contribute to vascular leakage and tissue damage (Glinton et al. 2017; Macheboeuf et al. 2011; Schmidt et al. 1984; Schmidt et al. 1987; Whitnack and Beachey 1985). To assess the ability of Mrp to interact with Fg fragments, we used SPR to evaluate the binding of Mrp to 200 nM FgD and 200 nM FgE. All nine Mrp bound to FgD but did not bind to FgE (Figure S3). The M1 protein and M53 protein were used as positive and negative controls, respectively (Figure S3). As expected, the M1 protein bound to FgD but not to FgE, while M53 did not interact with either FgD or FgE (Figure S3). We examined the interaction between Mrp and FgD across a concentration range of 0–200 nM to produce sensorgrams from which the *K*_*D*_ values were calculated (Figure 5 and Table 2). The *K*_*D*_ values for Mrp‐FgD interactions ranged from 51.6 to 94.1 nM, indicating a 4–18 fold lower affinity for FgD compared to full‐length Fg (Table 2).

### 2.5 Fibrinogen and plasminogen are recruited from human plasma by all Mrp

**FIGURE 6:**
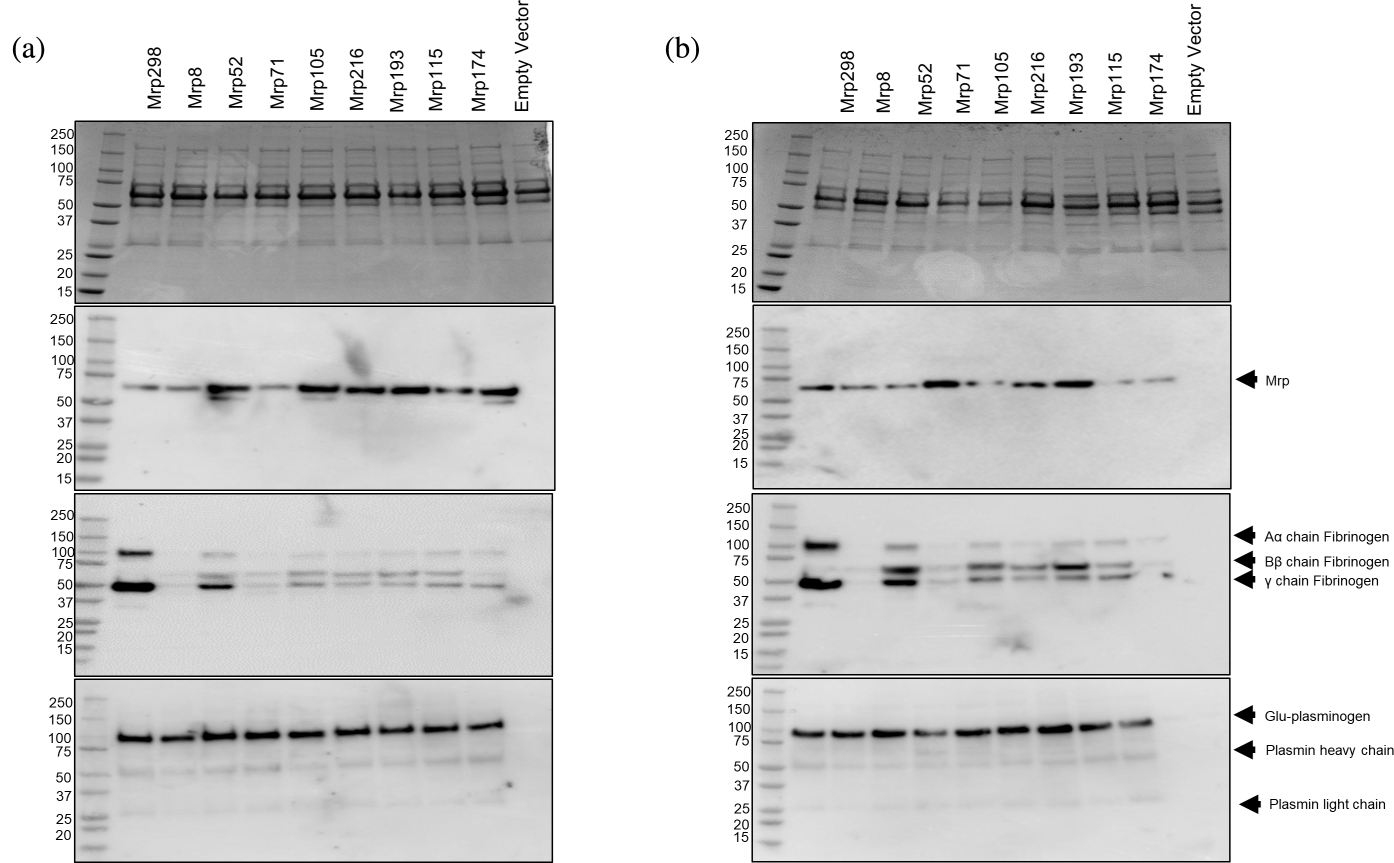
Interaction between Mrp and Fg in human plasma. Plasma pulldown assays were performed using NiNTA affinity chromatography on the lysates of BL21/DE3 *E. coli* expressing the nine 6x His‐tagged Mrp at (a) 25°C and (b) 37°C. The following plasma pulldown elutions were analyzed using 12% SDS‐PAGE stained with Coomassie blue (top panel) and Western blotting using α‐6x His‐tag antibody 27E8 for Mrp (second panel), Rabbit α‐Human Fg gamma pAb for Fg (third panel), or rabbit α‐human Plg (bottom panel). BL21/DE3 *E. coli* cells transformed with the empty vector (pGEX4T‐1 vector backbone without Mrp) were also examined in the pulldown assay as a control (empty vector).

Plasma pulldown assays were conducted to determine whether the two‐fold reduction in affinity for Fg observed with Mrp193 and Mrp216 affects their ability to acquire Fg in the presence of competing host proteins. Recombinant Mrp proteins were expressed in BL21/DE3 *Escherichia coli* cells and purified using Ni‐NTA affinity chromatography. Western blotting analysis, as shown in Figures 6 and S4, revealed co‐elution of Fg with all nine Mrp proteins studied (*n* = 3). These results confirm that all nine Mrp can acquire Fg from plasma at both 25 and 37°C, regardless of the number of FBDs or their affinity for Fg as determined by SPR.

Given that other Fg‐binding proteins on GAS, such as the M protein, are known to mediate the recruitment of Plg to the cell surface (McArthur et al. 2008; Sanderson‐Smith et al. 2012; Wang et al. 1995), we investigated whether Mrp can also recruit Plg. All Mrp successfully captured Plg from human plasma in pulldown assays conducted at both 25 and 37°C (Figure 6). Western blot analysis with an α‐Plg antibody detected bands at 60 and 25 kDa, corresponding to the heavy and light chains of Pln, respectively, suggesting that Mrp can mediate Pln acquisition.

### 2.6 Mrp does not directly bind Plg, but Mrp can bind Plg in the presence of Fg

**FIGURE 7:**
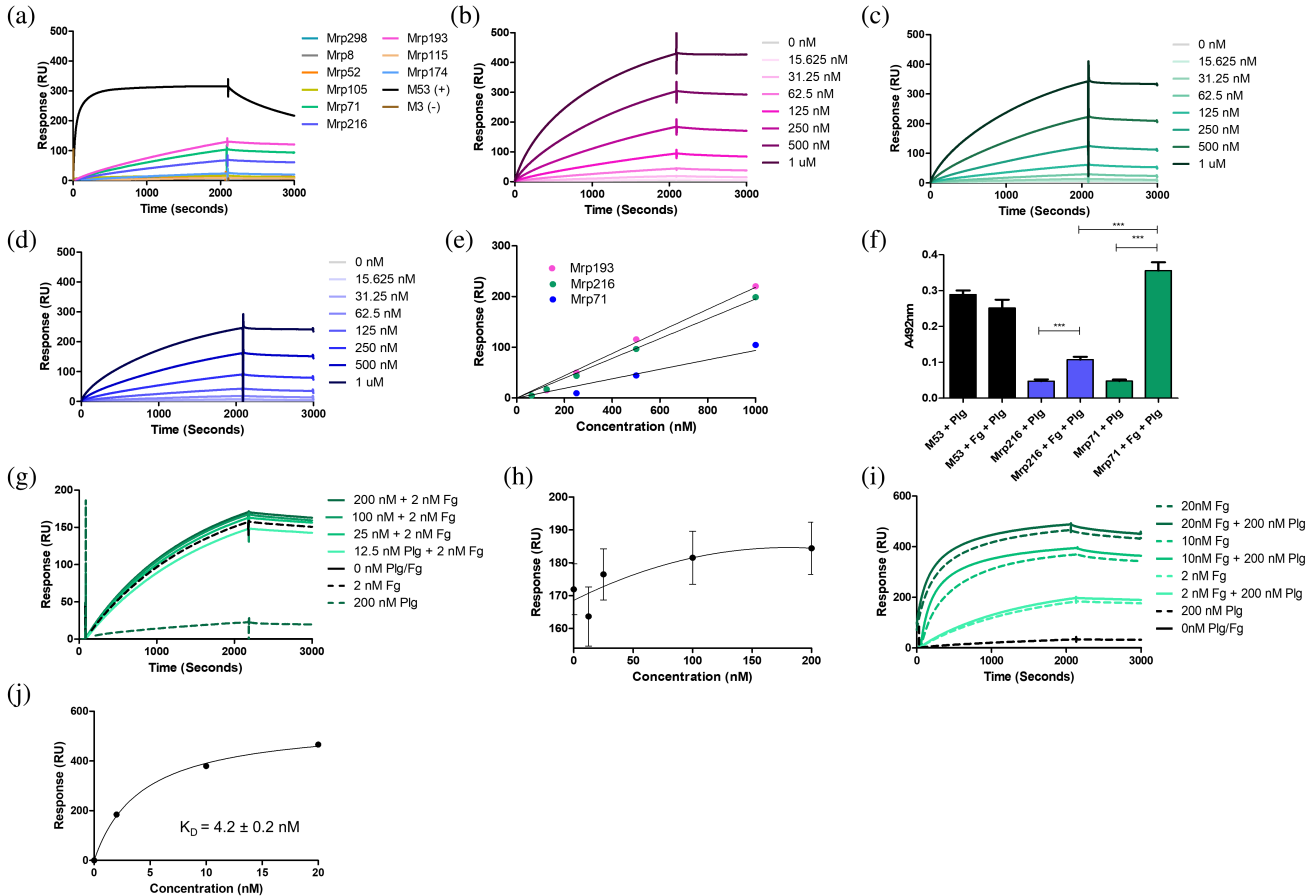
Binding between Mrp and Plg determined via surface plasmon resonance. (a) SPR sensorgram generated for nine genetically distinct Mrp and 200 nM Plg injected for 2100 s at a flow rate of 10 μL/min followed by a 900 s dissociation period. M53 (+) is a well characterized Plg‐binding protein, while M3 (−) has previously shown weak binding to Plg, thus they were used as controls for SPR experiments. Further characterization of the interaction between (b) Mrp193, (c) Mrp71, and (d) Mrp216 was conducted under the same experimental conditions. (e) Nonlinear fitting of sensorgrams establish that the interactions between Plg and Mrp193, Mrp71, and Mrp216, are weak interactions that did not reach saturation or show any sign of approaching plateau at the concentrations tested, indicating a *K*_*D*_ > 1 μM. (f) Plg binding assays examining the acquisition of Plg by Mrp in the presence or absence of Fg. Mean A492nm values (*n* = 3) were compared using one‐way ANOVA with post‐hoc analysis (Tukey multiple comparison tests). * indicates *p* ≤ 0.05, ** indicates *p* ≤ 0.01, *** indicates *p* ≤ 0.001. *p*‐value ≤0.05 was considered significant. (g) Characterization of the interaction between Mrp216 with increasing concentrations of Plg (0–200 nM) in the presence of 2 nM Fg were conducted using SPR. (h) Nonlinear fitting of sensorgrams confirm the formation of a tri‐molecular complex between Plg, Fg, and Mrp216. However, titrating Plg with a fixed concentration of Fg showed no change in response units, as binding reached a maximum capacity beyond which additional Plg did not increase binding. (i) Further characterization of the interaction between Mrp216 with a fixed concentration of Plg (200 nM) in the presence of increasing concentrations of Fg (0–20 nM) were conducted using SPR. (j) Examination of this data confirms the formation of a high affinity tri‐molecular complex between Plg, Fg, and Mrp216, with a nanomolar *K*_*D*_ .

The recruitment of host Plg by GAS can lead to severe tissue damage and excessive inflammation (Sanderson‐Smith et al. 2012). This damage is primarily due to the uncontrolled activation of Pln on the bacterial surface, which contributes to tissue destruction (McArthur et al. 2008; Sanderson‐Smith et al. 2012). To explore whether Mrp proteins can directly bind human Plg, we conducted SPR experiments (Figure 7). The M53 protein, known for its strong Plg binding, and the M3 protein, which does not bind Plg, were used as positive and negative controls, respectively (Sanderson‐Smith et al. 2014). As expected, M53 exhibited strong binding to Plg, while M3 did not interact with Plg at all (Figure 7a).

Among the Mrp proteins examined, only Mrp193, Mrp216, and Mrp71 showed any interaction with Plg (Figure 7a). However, the binding strengths of Mrp71 and Mrp216 to Plg were relatively weak, with dissociation constants (*K*_*D*_) greater than 1 μM, compared to the strong binding observed for M53 (*K*_*D*_ < 50 nM). Additionally, the interactions between Mrp and Plg did not reach a steady state, further indicating weak binding (Figure 7b–e) (Qiu et al. 2018; Qiu et al. 2019).

Fg‐binding M proteins interact indirectly with Plg by forming a tri‐molecular complex with Fg and Streptokinase type 2a (SK2a), which activates Pln (McArthur et al. 2008; Sanderson‐Smith et al. 2012; Wang et al. 1995). Alternative Fg‐binding receptors on GAS can also form a tri‐molecular Fg‐Plg‐Streptokinase type 1 (SK1) complex (Sanderson‐Smith et al. 2012; Wang et al. 1995). Given that Mrp binds Plg weakly, we performed solid‐phase microtitre assays to determine if Plg acquisition by Mrp could be enhanced in the presence of Fg (Figure 7f).

We examined Plg acquisition by Mrp216 from a GAS strain expressing SK type 2b and a Plg‐binding M protein (PAM) (Cook et al. 2012), and Mrp71 from a GAS strain expressing SK type 1 and lacking a PAM (McArthur et al. 2008). The results were compared to M53, a PAM that binds Plg directly and independently of Fg binding.

The data revealed low levels of Plg acquisition by Mrp216 and Mrp71 compared to M53 (*p* < 0.05). As expected, M53's Plg binding was unaffected by the presence of Fg. However, a significant increase in Plg acquisition was observed for both Mrp216 and Mrp71 in the presence of Fg (*p* < 0.05). Notably, Mrp71, derived from a PAM‐deficient strain, showed a greater increase in Plg acquisition with Fg compared to Mrp216, suggesting that Mrp‐Fg interactions may enhance Plg acquisition on the surface of PAM‐deficient strains expressing SK type 1.

Further analysis using SPR confirmed the formation of a molecular complex involving Mrp216, Plg, and Fg. Titration of plasminogen in the presence of a fixed concentration of Fg did not result in a difference in response units, with the interaction reaching a maximum binding capacity where additional Plg does not further increase binding (Figure 7g,h). This could be due to all Plg binding sites on Fg being occupied or the complex formation reaching a stable state. Conversely, measurement of complex interaction via titration of Fg in the presence of a fixed concentration of Plg revealed binding to Mrp at a *K*_*D*_ of 4.2 ± 0.2 nM, indicating a stronger binding affinity compared to the *K*_*D*_ value of 14.5 ± 2.2 nM observed for Mrp‐Fg interactions alone (Figure 7i,j and Table 2). Taken together, these data could suggest a cooperative binding mechanism where the presence of Plg enhances the binding affinity of Fg to Mrp, leading to a more stable tri‐molecular complex.

## 3 DISCUSSION

We investigated the interaction between Mrp and Fg, focusing on the functional implications of Mrp harboring one or two FBDs. Our findings show that having two FBDs does not result in a higher affinity of Mrp for Fg, nor does it enhance Mrp's ability to bind Fg in plasma. Notably, we demonstrate for the first time that Mrp indirectly recruits Plg through Mrp‐Fg interactions.

FgD, a fragment generated by the cleavage of Fg by Pln, plays a crucial role in amplifying Pln activation at infection sites through interactions with Plg‐SK complexes (Strickland et al. 1982; Váradi and Patthy 1983). Our study reveals that the binding of FgD is conserved across diverse Mrp. The acquisition of Plg and Pln at the GAS cell surface has been linked to tissue destruction and excessive stimulation of the inflammatory response during infection (Sanderson‐Smith et al. 2012). Together, these data highlight that Mrp is a significant virulence factor for GAS.

Examination of GAS genome sequences revealed that Mrp is present in 64.4% of *emm* types associated with global infections. Genetic diversity analysis identified four distinct clusters of Mrp (Frost 2020; Frost et al. 2020). In this study, we evaluated the presence of FBDs in a cohort of genetically diverse Mrp previously described (Proctor et al. 2024). To assess the conservation and number of FBDs, we computed pairwise identities between the previously published Mrp4 sequence (Li and Courtney 2011) and the nine genetically diverse Mrp examined. We found that FBD2 was present in all nine Mrp. Notably, the sequence NENAQLKEAIEGYVQ within Mrp4, which is critical for Fg binding (Li and Courtney 2011), was 100% conserved across all Mrp in this study. In contrast, the FBD1 sequence was only present in Mrp from cluster four.

The role of the additional FBD in Mrp remains unclear, as both FBD1 and FBD2 have been shown to mediate Fg binding (Li and Courtney 2011). FBD2 peptides have been shown to be more effective than FBD1 in inhibiting Fg binding to GAS in competitive binding assays (Li and Courtney 2011). Despite this, both FBD1‐ and FBD2‐peptides are able to inhibit the growth of GAS in human blood by more than 80% (Li and Courtney 2011). To investigate whether Mrp with two FBDs, a feature unique to cluster four, exhibit greater affinity for Fg compared to Mrp with a single FBD, we analyzed binding to nine Mrp. Each Mrp bound Fg with high affinity, ranging from 5.77 to 14.5 nM. Notably, Mrp193 and Mrp216, both from cluster four, showed a twofold lower affinity for Fg compared to the other Mrp in this study. There were no significant differences in the FBD2 amino acid sequences between Mrp193, Mrp216, and the other Mrp, suggesting that the observed differences in affinity may be attributed to variations in the FBD1 sequence.

Examination of the FBD1 amino acid sequences in Mrp193 and Mrp216 revealed differences in the N‐terminal region compared to Mrp115 and Mrp174. Specifically, the helix‐breaking prolines and glycine's present in Mrp193 and Mrp216 are replaced by hydrophobic amino acids in Mrp115 and Mrp174.

Prolines are known to flank the ends of helical proteins and restrict transient residual helicity, contributing to various degrees of conformational plasticity and influencing the affinity for partner proteins (Crabtree et al. 2017). For example, prolines flanking helices in the disordered transactivation domain of p53 have been shown to increase peak residual helicity and result in a tenfold reduction in affinity for its binding partner, MDM2 (Borcherds et al. 2014; Crabtree et al. 2017). Similarly, studies on the intrinsically disordered transactivation domain of the myeloblastosis oncoprotein c‐Myb and its ordered binding partner KIX have demonstrated that incorporating glycine and proline into c‐Myb mutants reduces binding affinity to KIX (Poosapati et al. 2018). Therefore, it is plausible that the reduced affinity of Mrp193 and Mrp216 for Fg may be attributed to the presence of helix‐flanking prolines, which could lead to conformational changes in the FBD1. Further studies involving the mutation of these prolines to helix‐stabilizing amino acids are needed to confirm this hypothesis.

Despite the lower binding affinity of Mrp193 and Mrp216 for Fg, our study confirms that high‐affinity Fg‐binding is conserved across a selection of genetically diverse Mrp. Plasma pulldown assays further demonstrate that all nine Mrp can acquire Fg from human plasma, although to varying extents, regardless of the number of FBDs or their affinity for Fg. The stability of a protein complex is influenced by the binding affinity, while specificity is governed by the discriminant binding of each partner to other competing biomolecules (Yan and Wang 2012). The findings of this study, along with previous work (Proctor et al. 2024), demonstrate that the nine Mrp examined can concurrently acquire Plg, Fg, and IgG. The potential for IgG‐Mrp interactions to either inhibit or enhance the binding of Fg or Plg is a significant consideration, particularly since α‐helical coiled‐coil proteins, like those described by Ermert et al. (2019), have been shown to enhance the binding of other host proteins. In that study, IgG bound via Fc to protein H (an M‐like protein) enhanced the recruitment of C4BP. Future studies should consider investigating this potential competitive or cooperative binding phenomenon further.

Interestingly, Mrp193 and Mrp216 are expressed by GAS strains belonging to the D4 *emm* cluster, whereas the other Mrp tested are associated with GAS strains from the E2, E3, E4, and E6 *emm* clusters (Sanderson‐Smith et al. 2014). Diversity within the M protein family has been suggested to have functional implications, providing GAS with several alternate mechanisms to achieve virulence in the host (Frost 2020; Frost et al. 2018; Smeesters et al. 2010). For example, M proteins from the D4 cluster are known to bind Plg, while those from the E2, E3, E4, and E6 clusters interact with IgG, IgA, albumin, and/or C4BP (Sanderson‐Smith et al. 2014). Therefore, the varying extents of Fg acquisition by Mrp observed in the current study may relate to the expression of other members of the M protein family; however, further investigation with a larger dataset of Mrp from both clusters D and E is required in future studies to validate this hypothesis. While the data suggest that Mrp from the D4 cluster exhibit lower affinity for Fg, all Mrp tested demonstrate the ability to bind Fg in the presence of other plasma proteins, suggesting an important role for Mrp‐mediated Fg binding by GAS.

An advantage of proteins containing multiple binding domains for a single ligand is their potential for functional plasticity, including the possibility of allosteric interactions (Bhaskara and Srinivasan 2011). Allosteric effects have been observed in the M protein family. For example, the binding of albumin or IgG to purified M1 protein significantly enhances Fg binding (Cedervall et al. 1995). Additionally, IgG binding to Protein H (Ermert et al. 2019) and Enn (Frost 2020) has been shown to increase the recruitment of C4BP to the bacterial surface. Furthermore, M protein binding to Fg enhances Plg acquisition at the GAS cell surface (Glinton et al. 2017).

In the current study, SPR and plasma pulldown assays revealed that FBD1 is not essential for Fg binding and does not significantly affect the affinity of Mrp for Fg. This suggests that FBD1 may not serve as an allosteric site where the binding of one plasma protein enhances the binding of another. This is supported by our finding that Mrp71, which contains only FBD2, demonstrated greater Fg‐augmented Plg binding compared to Mrp216, which contains both FBD1 and FBD2. Although Mrp binds to Plg with relatively weak affinity, pre‐incubation with Fg significantly increased Plg acquisition by Mrp, a phenomenon previously observed for the M protein, which facilitates Pln activation at the GAS cell surface (Glinton et al. 2017). SPR data suggest that Plg enhances the binding of Fg to Mrp through a cooperative interaction, resulting in a stronger binding affinity when both are present compared to Fg alone. Plg may stabilize the binding of Fg to Mrp or form a more stable complex with Mrp and Fg. Overall, the presence of Plg enhances the binding affinity of Fg to Mrp, leading to a stable tri‐molecular complex.

Pln cleaves Fg into FgD and FgE, with the interaction between M protein and Fg mediated by the D domains of Fg (Glinton et al. 2017; Schmidt et al. 1984; Schmidt et al. 1987; Whitnack and Beachey 1985). The M‐FgD interaction is known to inhibit the opsonization of GAS (Whitnack and Beachey 1985). Specifically, the M1‐FgD interaction facilitates the formation of supramolecular networks between the M1 protein and Fg, leading to a pro‐inflammatory response that results in vascular leakage and tissue injury, key features of streptococcal toxic shock syndrome (STSS) (Macheboeuf et al. 2011). Moreover, the recruitment of FgD at infection sites enhances Pln recruitment to the GAS cell surface, initiating the release of products that affect blood vessel permeability and promote the accumulation of inflammatory cells (Castellino and Ploplis 2005; Danø et al. 1985; Strickland et al. 1982; Váradi and Patthy 1983).

Interactions between the M protein and FgD have been previously shown to facilitate the subsequent binding of human Plg to FgE (Glinton et al. 2017). The data presented in this study demonstrate that, like the M protein, Mrp binds to Fg via the FgD domain, with an affinity for FgD that is 4–18 times lower than that for full‐length Fg. This finding aligns with previous research on the M protein, which showed that the average affinity of the M protein for FgD is approximately 30 times lower than for intact Fg (Whitnack and Beachey 1985). The reduced affinity observed in our assay with purified FgD fragments may be attributed to their lower avidity compared to the full‐length Fg molecule, which contains two FgD fragments.

Previous studies suggest Fg‐D binding motifs vary between M proteins. This is because M proteins bind to Fg via the B‐repeats, which are highly variable between M protein types (Glinton et al. 2017). The conservation of Fg‐binding in all Mrp suggests that FBD2 alone is sufficient to mediate binding by diverse Mrp. While the amino acid sequence of the M1 B‐repeats and FBD2 of Mrp share low sequence homology (18.4%), they exhibit conformational similarities. Both the B‐repeat of M1 and FBD2 adopt an α‐helical conformation, with hydrophobic amino acids occupying the a and d positions of the heptad repeats (Glinton et al. 2017).

To date, the interaction between Mrp and Fg has been characterized in a single isolate with two FBDs (Li and Courtney 2011). While it was hypothesized that Mrp‐Fg binding was common to all Mrp based on sequence analysis, this had not been confirmed (Courtney et al. 2006; Courtney and Li 2013). In this study, we performed a functional characterization using a genetically diverse collection of Mrp and found that the presence of two fibrinogen binding domains within Mrp does not enhance the affinity for Fg. Additionally, the presence of secondary binding sites did not provide Mrp with an advantage in acquiring Fg under the physiologically relevant conditions of human plasma.

High specificity for Fg was confirmed in the FBDs of Mrp, as Fg acquisition was detected across all genetically diverse Mrp in plasma assays. The FgD domain of Fg was shown to facilitate Mrp‐Fg interactions. These findings confirm that the interaction between Mrp and Fg is conserved, suggesting that the acquisition of Plg in the Fg‐Plg‐Mrp complex is also conserved. Despite sequence diversity among strains, these data indicate that the role of Mrp in GAS virulence is maintained, highlighting Mrp as a crucial mediator of host–pathogen interactions during GAS infection.

## 4 METHODS

### 4.1 Ethics statement

All experiments involving the use of human plasma were conducted with informed consent of healthy volunteers, approved, and authorized by the University of Wollongong Human Research Ethics Committee (Protocol HE08/250).

### 4.2 Mass photometry

The oligomerisation state of Mrp was evaluated using mass photometry. Samples with 100 nM Mrp in PBS were prepared, and 10 μL of each sample was analyzed for 1 min at a rate of 600 frames per minute using an ONEMP mass photometer (Refeyn LTD). Mass photometry experiments were conducted in triplicate. Data were collected and analyzed with the AcquireMP and DiscoverMP software, version 1.2.3 (Refeyn LTD), as per (Cole et al. 2017).

### 4.3 Negative staining transmission electron microscopy

Five microliter aliquots of each Mrp at 50 μg/mL was applied to glow discharged carbon grids (TedPella) for 1 min and washed in Milli‐Q® water for 30 s. The grids were then negatively stained with 2% (w/v) uranyl acetate, blotted, air‐dried, and imaged using an FEI Tecnai T12 transmission electron microscope with a Gatan Rio 4 camera (at the Cryogenic Electron Microscopy Facility, Molecular Horizons, University of Wollongong). Lengths of the Mrp molecules were measured using Image J (Schneider et al. 2012). Average fibril lengths ± SEM were calculated and charted (*n* = 100 for each sample) using GraphPad Prism 5 (GraphPad Software). A one‐way ANOVA was used to determine any significant difference between the lengths of Mrp examined and determined no significant difference between all Mrp, with a *p*‐value ≤0.05 considered significant.

### 4.4 Amino acid sequence analysis of Mrp fibrinogen binding domain

The FBD1 and FBD2 of 221 unique Mrp amino acid sequences identified from 1668 GAS genomes by Frost et al. (2023) were performed using default settings in Geneious (version 6.0, Biomatters). The Nine Mrp in this study were previously selected in Proctor et al. (2024) to represent the genetic diversity of the Mrp family. A pairwise MUSCLE alignment was produced for the amino acids in the FBD1 and FBD2 sequence (Li and Courtney 2011) of Mrp4 using default settings in Geneious (version 6.0, Biomatters).

### 4.5 Protein purification

Nine *mrp* genes were subcloned into pGEX4T1 (Invitrogen) via *Bam*HI and *Eco*RI restriction enzyme sites by Genscript to generate N‐terminal glutathione S‐transferase (GST) fusion proteins without the N‐terminal signal peptide and C‐terminal cell wall anchoring domains (Proctor et al. 2024). Additionally, the *mrp* genes incorporated a C‐terminal hexahistidine tag (6x His‐tag) and stop codon (TAA) to facilitate the uniform orientation of all Mrp upon capture onto a sensor surface in SPR experiments and mimic the in vivo orientation of Mrp on the GAS cell surface (Podbielski et al. 1994). The inclusion of both a 6x His‐tag and a GST tag on the recombinant protein enabled purification as previously described by Proctor et al. (2024). Briefly, the nine recombinant Mrp variants were expressed in *E. coli* BL21/DE3 cells transformed with pGEX4T1‐*mrp*, and the overexpression of Mrp was induced with 0.1 mM isopropyl β‐D‐1‐thiogalactopyranoside (Sigma‐Aldrich) at 30°C for 4 h with agitation. Cell lysates were prepared in ice‐cold phosphate‐buffered saline (PBS) containing 1% Triton X‐100 (Sigma‐Aldrich), 2 μg/mL DNase I (Sigma‐Aldrich), 10 μM phenylmethylsulfonyl fluoride (Sigma‐Aldrich), and 1 mg/mL lysozyme (Astral Scientific) using the Branson Sonifier 250 (Emerson) at 30% duty cycle with the microtip output control 2 for 2 min (10 s on, 10 s off). Cell lysates were filtered with a 0.22‐μM filter (Millipore) after centrifugation at 12,000*g* for 10 min to remove cellular debris before being applied to a GST‐agarose column (Sigma‐Aldrich) with a bed volume of 1 mL. Protein was eluted with glutathione elution buffer (10 mM reduced glutathione (Sigma‐Aldrich) in 50 mM Tris–HCl, pH 8.0) and incubated with 60 U of thrombin (Haematologic Technologies) for 4 h at room temperature to cleave the GST tag from the recombinant Mrp. Cleaved Mrp was incubated with a 0.5‐mL Ni‐NTA column (Qiagen) for 1 h, and recombinant protein was eluted using native elution buffer (NaCl 17.5 g/L, NaH_2_PO_4_ 6.9 g/L, and Imidazole 17 g/L in Milli‐Q® water) and dialyzed into PBS.

### 4.6 Surface plasmon resonance

Binding interactions were analyzed using surface plasmon resonance (SPR) on a BIAcore T200 (GE Healthcare). Recombinant 6x His‐tagged Mrp were immobilized onto Series S Sensor Chip NTA (GE Healthcare) and binding to Human Fg (F4883, Sigma‐Aldrich), Human FgD (RP‐43143, Invitrogen), Human FgE (RP‐43144, Invitrogen), and human Glu‐Plg (HCPG‐0130, Haematologic Technologies Inc.) was determined at 25°C on the BiacoreT200 via steady‐state mode. SPR experiments were conducted as per the methods described in Proctor et al. (2024) except for the following alterations. The concentration range examined for each analyte over a series of seven separate injections, the injection time and rate, as well as the dissociation time for each analyte are summarized in Table 3. A steady‐state affinity plot was used to determine the *K*_*D*_ of the interaction between Mrp and each analyte. Data was analyzed via nonlinear fitting of the sensorgrams according to a 1:1 Langmuir binding model in BIA evaluation *version* 4.0.1 (Biacore AB, Uppsala, Sweden) and Scatchard plot analysis in GraphPad Prism *version* 5 (GraphPad Software, California) (Scatchard 1949). Data presented represent the average of *n* = 3 ± SEM.

**TABLE 3:** Summary of SPR methods used to characterize the interaction between Mrp and human serum proteins.

Host protein	Concentration range (nM)	Injection time (s) and rate (μL/min)	Dissociation time (s)
Fg	0–200 nM	2700 s at 5 μL/min	900 s
FgD	200 nM^a^	60 s at 5 μL/min^a^	90 s^a^
	0–200 nM^b^	2700 s at 5 μL/min^b^	900 s^b^
FgE	200 nM^a^	60 s at 5 μL/min^a^	90 s^a^
Glu‐Plg	0–1000 nM	2100 s at 5 μL/min	900 s

^a^ SPR conditions used for the initial screening of the Mrp binding domain on Fg.

^b^ After initial screening analysis, FgD was further characterized under these conditions.

### 4.7 Protein structure modeling with AlphaFold

The AlphaFold.ipynb Colab notebook was used to predict the structure of dimeric Mrp using a slightly simplified version of AlphaFold V2.3.2 (https://colab.research.google.com/github/deepmind/alphafold/blob/main/notebooks/AlphaFold.ipynb, accessed on 10 January 2023) (Evans et al. 2022; Jumper et al. 2021). Molecular graphics and visual analyses of the Coulombic electrostatic potential for PBD files generated in AlphaFold were performed with UCSF ChimeraX 1.3 (Meng et al. 2023).

### 4.8 EMBOSS: Charge

The electrostatic potential of the unstructured region of the FBD1 region in Mrp was predicted using EMBOSS: charge (Rice et al. 2000). Charge values were obtained using the EMBOSS Charge with a window size of five amino acids. The predictions were plotted in GraphPad Prism 5.

### 4.9 Plasma pulldown assay and western blotting

Interactions between recombinant 6x His‐tagged Mrp and Fg or Plg in human plasma were analyzed using pulldown assays as previously described at 37°C (Proctor et al. 2024). Interactions between Mrp and serum proteins were detected via immunoblotting onto PVDF membranes (Bio‐Rad) after 8 μg total protein was separated on a 4%–20% Mini‐PROTEAN® TGX Stain‐Free™ SDS‐PAGE gel (Bio‐Rad). Membranes were blocked for 1 h with 5% skim milk in Tris‐Buffered Saline containing 0.1% (v/v) Tween 20 (TBST) and then incubated for an hour with 1:1000 mouse α‐6x His‐tag antibody 27E8 (#2336, Cell Signalling Technology) for Mrp, 1:1000 Rabbit α‐Human Fg gamma pAb (PA5‐95397, Invitrogen) for Fg, or 1:5000 rabbit α‐human Plg (Calbiochem) for Plg. Membranes were then washed thrice with TBST for 5 min between antibody incubations. Membranes incubated with the mouse α‐6x His‐tag antibody were then incubated for 1 h at RT with 1:30,000 goat α‐mouse IgG conjugated with horseradish peroxidase (HRP) (ab97023, Abcam). Membranes incubated with the rabbit α‐human Plg or rabbit α‐human Fg gamma pAb were then incubated for 1 h at RT with 1:3000 Goat α‐rabbit IgG HRP conjugate (#65‐6120, Invitrogen). Blots were processed with Clarity Max Western ECL Blotting Substrate (Bio‐Rad) and imaged on the Amersham AI600 (GE Healthcare, Chicago). BL21/DE3 *E. coli* cells transformed with the empty vector (pGEX4T‐1 vector backbone without *mrp*) were also examined in the pulldown assay as a control and referred to as empty vector. Western blots were performed in triplicate at both 25 and 37°C.

### 4.10 Plasminogen binding assay

Plates were coated with either Mrp216, Mrp71, or M53 (Plg‐binding M protein; PAM control) diluted to a final concentration of 5 μg/mL in 1M NaHCO_3_ pH 9.6 and incubated overnight at 4°C. Plates were blocked with 5% skim milk in 1X PBS‐0.1% Tween20 (PBST) for 1 h at RT to prevent non‐specific binding and washed with PBST 5 times before plates were treated with 100 nM Fg prepared in 1% skim milk in 1X PBS for 1 h at RT. Plates were subsequently washed again 5 times with PBST and incubated with 100 nM biotinylated‐Plg in 1% skim milk in 1X PBS for 1 h at RT. Plates were again washed 5 times with PBST and incubated with 1:1000 Streptavidin‐HRP (SA‐HRP) for 1 h at RT. Plates were washed 5 times with PBST before detection was performed with peroxidase substrate solution (8 mM Na_2_HPO_4_, pH 5.0, 2.2 mM o‐phenylenediamine, 3% H_2_O_2_) for 15 min, and the reaction was stopped with 1M HCl and read on a SPECTROstar Nano Microplate Reader (BMG labtech, Victoria, Australia) at 490 nm.

## AUTHOR CONTRIBUTIONS

**Emma‐Jayne Proctor:** Conceptualization; methodology; investigation; formal analysis; writing – original draft. **Hannah R. Frost:** Methodology; investigation. **Bhanu Mantri:** Methodology; investigation. **Sandeep Satapathy:** Methodology; investigation. **Gwenaëlle Botquin:** Methodology. **Jody Gorman:** Supervision. **David M. P. De Oliveira:** Writing – review and editing; resources. **Jason McArthur:** Supervision; writing – review and editing; resources. **Mark R. Davies:** Writing – review and editing; resources. **Gökhan Tolun:** Writing – review and editing; resources; methodology; supervision. **Anne Botteaux:** Conceptualization; writing – review and editing. **Pierre Smeesters:** Writing – original draft; conceptualization. **Martina Sanderson‐Smith:** Conceptualization; writing – review and editing; resources; funding acquisition; supervision.

## CONFLICT OF INTEREST STATEMENT

The authors declare no conflicts of interest.

## Supporting information


**Figure S1.** Confirmation that Mrp is expressed as a dimer using mass photometry.
**Figure S2.** Binding between M1 and M53 controls with Fg determined via SPR.
**Figure S3.** Screening binding between Mrp and Fg Fragment D (FgD) and Fragment E (FgE) determined via surface plasmon resonance.
**Figure S4.** Additional replicates of plasma pulldown assays.

## Data Availability

The data that support the findings of this study are available in Supporting Information of this article.
