# Supplementary material for: Fibrinogen‐binding M‐related proteins facilitate the recruitment of plasminogen by Streptococcus pyogenes
Source: Protein Sci. 2025 Mar 18;34(4):e70078. doi: 10.1002/pro.70078 (PMC11917135; doi:10.1002/pro.70078)
Supplement: Supplementary file 1 — Figure S1. Confirmation that Mrp is expressed as a dimer using mass photometry. Figure S2. Binding between M1 and M53 controls with Fg determined via SPR. Figure S3. Screening binding between Mrp and Fg Fragment D (FgD) and Fragment E (FgE) determined via surface plasmon resonance. Figure S4. Additional replicates of plasma pulldown assays. [file PRO-34-e70078-s001.docx]

# SUPPLEMENTARY MATERIAL

# Fibrinogen-binding M-related proteins facilitate the recruitment of plasminogen by *Streptococcus pyogenes*

Emma-Jayne Proctor ^a^, Hannah R. Frost ^b^, Bhanu Mantri ^a^, Sandeep Satapathy ^a,c^, Gwenaëlle Botquin^b^, Jody Gorman^a^, David M. P. De Oliveira^d^, Jason McArthur ^a^ Mark R. Davies ^e^, Gökhan Tolun ^a^, Anne Botteaux ^b^, Pierre Smeesters ^b^, and Martina Sanderson-Smith ^a*^

^a^Molecular Horizons Research Institute and School of Chemistry and Molecular Bioscience, University of Wollongong, Wollongong, Australia

^b^Molecular Bacteriology Laboratory, European Plotkins Institute for Vaccinology (EPIV), Université Libre de Bruxelles, Brussels, Belgium

^c^ The Broad Institute of MIT and Harvard, Cambridge, Massachusetts.

^d^ The Institute for Molecular Biosciences, Centre for Superbug Solutions, The University of Queensland, QLD, Australia.  ^e^ Department of Microbiology and Immunology, at the Peter Doherty Institute for Infection and Immunity, The University of Melbourne, Victoria, Australia.

## List of Supplementary Material in this document:

1. Sup Fig 1 - Confirmation that Mrp is expressed as a dimer using Mass Photometry.
2. Sup Fig 2 - Binding between M1 and M53 controls with Fg determined via SPR.
3. Sup Fig 3 - Screening binding between Mrp and Fg Fragment D (FgD) and Fragment E (FgE) determined via surface plasmon resonance.
4. Sup Fig 4 - Additional replicates of Plasma pulldown assays.

###
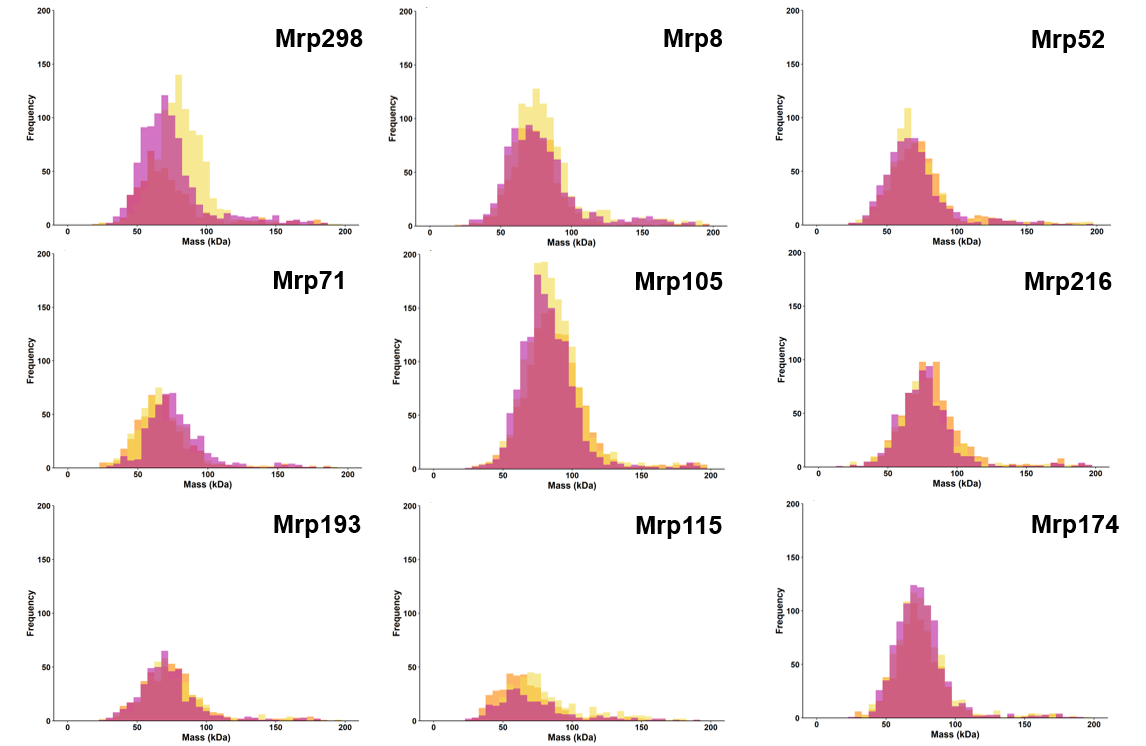


### **Sup Fig 1: Confirmation that Mrp is expressed as a dimer using Mass Photometry.** The oligimerisation state of all Mrp in this study were examined using Mass photometry diluted to a final concentration of 100nM in PBS. Monomeric Mrp (35-40kDa), which was absent from all spectra, while the Dimeric Mrp (70-80kDa) was the sole species present for all Mrp in this study. Three curves shown for each protein depict n=3 replicates.


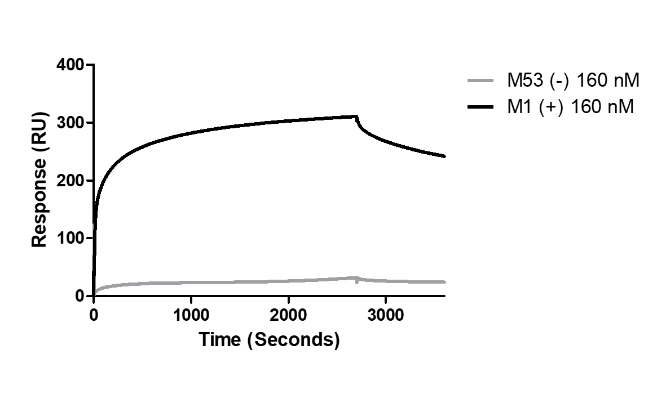


### **Sup Fig 2: Binding between M1 and M53 controls with Fg determined via SPR.** M1 (+) is a well characterised Fg-binding protein, while M53 (-) has previously shown weak binding to Fg, thus they were examined as controls for SPR experiments. Recombinant poly-histidine tagged M1 or M53 was immobilised onto Series S Sensor Chip NTA (GE Healthcare) at 40 RU and binding to 160nM Fg was determined on the BiacoreT200 for 2800s at a flow rate of 5 μl/min followed by a 900s dissociation period.

### **
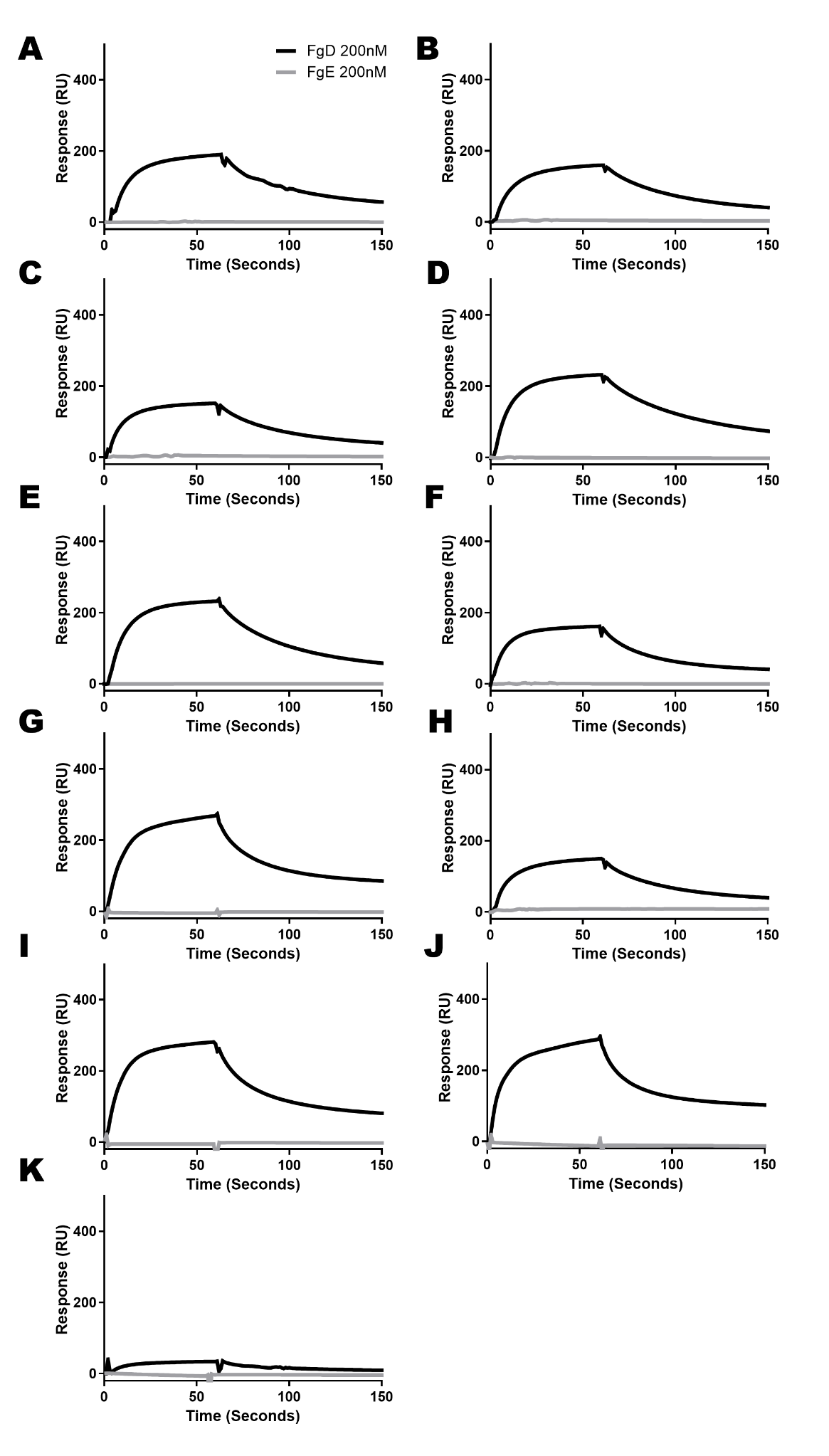
 Sup Fig 3: Screening binding between Mrp and Fg Fragment D (FgD) and Fragment E (FgE) determined via surface plasmon resonance.** Binding to 200 nM FgD and 200 nM FgE was determined on the BiacoreT200 for 60s at a flow rate of 5 μl/min followed by a 90s dissociation period. . (A) Mrp298, (B) Mrp10, (C) Mrp52, (D) Mrp71, (E) Mrp105, (F) Mrp216, (G) Mrp 210.5, (H) Mrp115, and (I) Mrp174. Binding between M1 and M53 controls with Fg Fragment D (FgD) and Fragment E (FgE) were also determined via surface plasmon resonance. M1 (+) is a well characterised Fg-binding protein, while M53 (-) has previously shown weak binding to Fg, thus they were examined as controls for SPR experiments. Recombinant poly-histidine tagged (J) M1 or (K) M53 was immobilised onto Series S Sensor Chip NTA (GE Healthcare) at 40 RU and binding to 200nM FgD and 200nM FgE binding was determined on the BiacoreT200 for 60s at a flow rate of 5 μl/min followed by a 90s dissociation period.

###
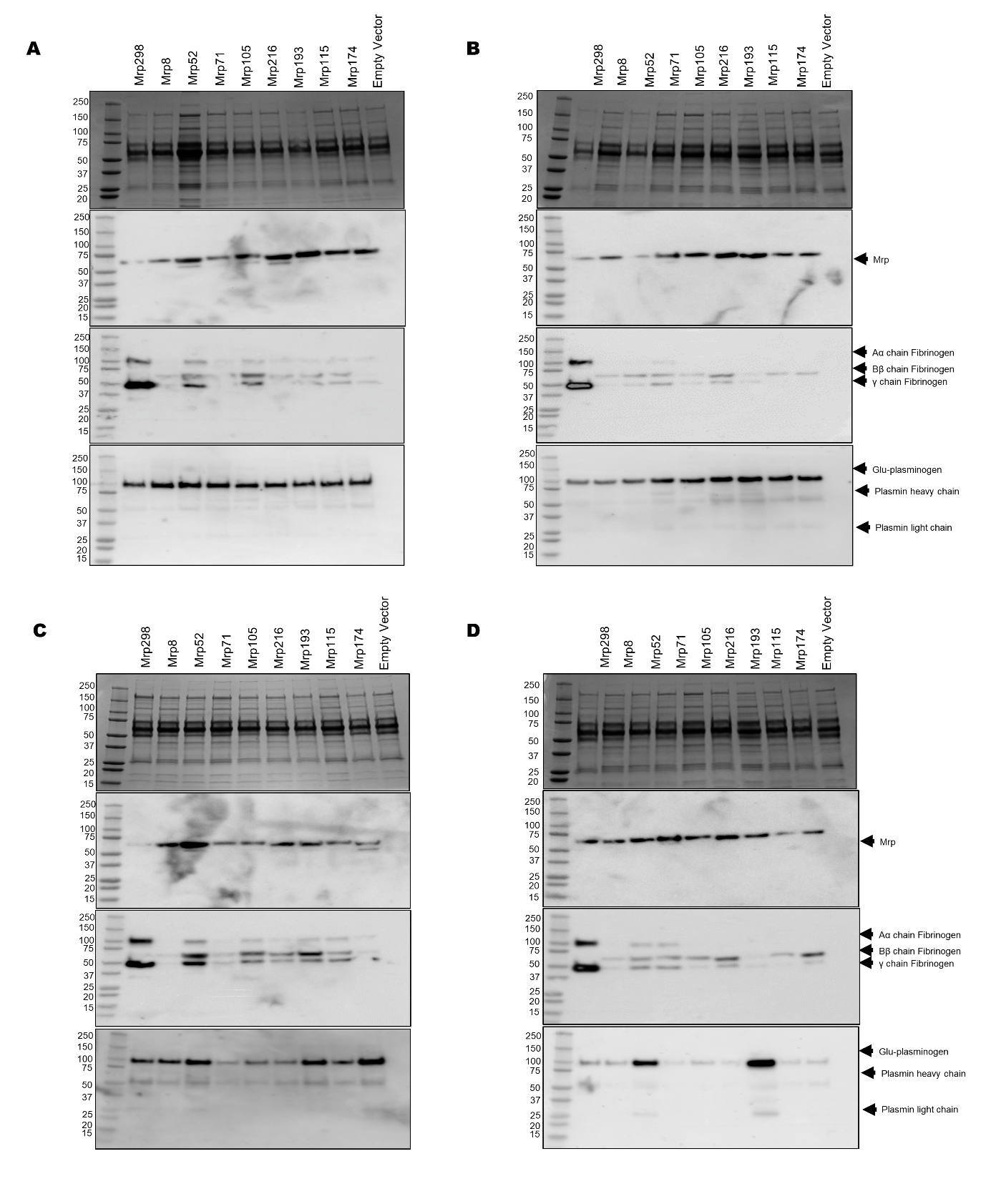


### **Sup Fig 4: Interaction between Mrp and Fg in human plasma.** Plasma pulldown assays were performed using NiNTA affinity chromatography on the lysates of BL21/DE3 *E. coli* expressing the nine 6 x his-tagged Mrp. Replicate two of this assay at **A)** 25°C and **B)** 37°C. Replicate three of this assay at **C)** 25°C and **D)** 37°C. The following plasma pulldown elutions were analysed using 12% SDS-PAGE stained with Coomassie blue (top panel) and Western blotting using α-6x His-tag antibody 27E8 for Mrp (Second panel), Rabbit α-Human Fg gamma pAb for Fg (third panel), or rabbit α-human Plg (bottom panel). BL21/DE3 *E coli* cells transformed with the empty vector (pGEX4T-1 vector backbone without Mrp) were also examined in the pulldown assay as a control (empty vector).
